# CD95 Structure, Aggregation and Cell Signaling

**DOI:** 10.3389/fcell.2020.00314

**Published:** 2020-05-05

**Authors:** Nicolas Levoin, Mickael Jean, Patrick Legembre

**Affiliations:** ^1^Bioprojet Biotech, Saint-Grégoire, France; ^2^Univ Rennes, CNRS, ISCR-UMR 6226, Rennes, France; ^3^INSERM U1262, CRIBL, Université de Limoges, Limoges, France

**Keywords:** aggregation, apoptosis, Fas, inflammation, migration, stoichiometry

## Abstract

CD95 is a pre-ligand-associated transmembrane (TM) receptor. The interaction with its ligand CD95L brings to a next level its aggregation and triggers different signaling pathways, leading to cell motility, differentiation or cell death. This diversity of biological responses associated with a unique receptor devoid of enzymatic property raises the question of whether different ligands exist, or whether the fine-tuned control of CD95 aggregation and conformation, its distribution within certain plasma membrane sub-domains or the pattern of post-translational modifications account for this such broad-range of cell signaling. Herein, we review how the different domains of CD95 and their post-translational modifications or the different forms of CD95L can participate in the receptor aggregation and induction of cell signaling. Understanding how CD95 response goes from cell death to cell proliferation, differentiation and motility is a prerequisite to reveal novel therapeutic options to treat chronic inflammatory disorders and cancers.

## Introduction

**FIGURE 1:**
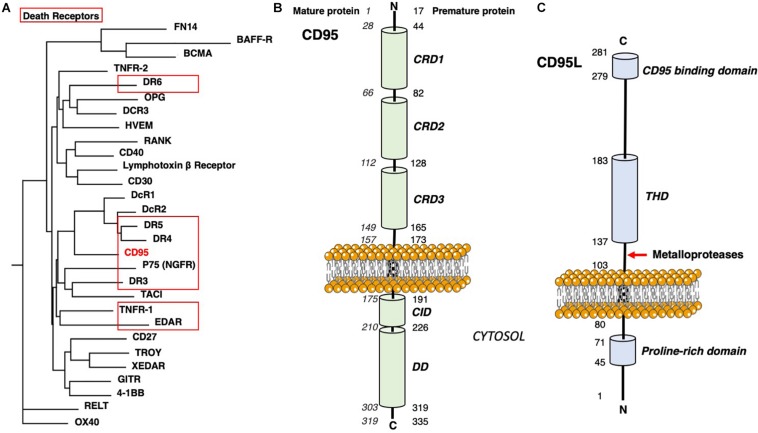
CD95 domains. **(A)** Phylogenetic tree of human TNF receptors. DD-containing receptors are surrounded in red. Sequences were aligned with MAFFT 7 (Katoh and Standley, 2013) following L-INS-I strategy with BLOSUM 30 matrix, incorporating Mafft homologs and bootstrapping. MaxAlign option was used to increase the number of gap-free sites. The tree was built with Phylip 3.6 (Felsenstein, 1989) using a Neighbor-Joining method. **(B)** Main domains in the CD95 protein. **(C)** Main domains in the CD95L protein.

Many Tumor necrosis factor (TNF) receptor superfamily members display significant roles in the progression of human diseases, such as the death domain (DD)-containing receptors including CD95, TNF-related apoptosis-inducing ligand receptor (DR4 and DR5), TNFR1, DR3, DR6, nerve growth factor receptor (NGFR), and ectodysplasin receptor (EDAR, Figure 1A). These receptors are characterized by the presence of an intracellular DD, which is required for their apoptosis-inducing activity (Dostert et al., 2019). Several of them, including CD95 and TNFR1, are known to form multimers not only in the presence but also in the absence of their cognate trimeric ligands (Chan et al., 2000; Siegel et al., 2000), rendering complex to determine the nature and role of their aggregation in the cell signaling process. This review discusses how the CD95 stoichiometry is controlled by receptor-dependent and independent processes, and how stoichiometry can affect the implementation of apoptotic or non-apoptotic signals.

## A Unique CD95 Receptor but at Least Two Forms of the Ligand

### CD95

CD95 (also known as Fas, Apo-1, TNFRSF6) is a 319 amino acid type I transmembrane glycoprotein (Itoh et al., 1991; Figure 1B). In the presence of its ligand CD95L, the receptor interacts with the adaptor protein Fas-associated protein with death domain (FADD) through homotypic DD-mediated interactions. FADD in turn recruits the protease caspase-8 and the long form of the regulator of apoptosis cellular FADD-like interleukin-1-β-converting enzyme-inhibitory protein (cFLIP_L_) *via* death effector domain (DED) homotypic binding. Together, these proteins form a complex designated DISK for death-inducing signaling complex (Kischkel et al., 1995). The initial steps of CD95-DISK formation are quite well defined and some of them are shared with other death receptors of the TNFR superfamily.

Although initially described as a pure death receptor, CD95 undergoes a paradigm change, which might lead to a therapeutic revolution. Indeed, cumulative evidence support that CD95 is not only able to trigger a cell death signal but can also promote inflammation and normal and tumor cell growth and migration through the implementation of non-apoptotic cellular functions including PI3K, NFkB, and JNK MAPKs (Desbarats and Newell, 2000; Desbarats et al., 2003; Kleber et al., 2008; O’ Reilly et al., 2009; Hoogwater et al., 2010; Tauzin et al., 2011; Gao et al., 2016; Poissonnier et al., 2016). Members of DISK including FADD and caspase-8 could also participate in the induction of these non-apoptotic cell signaling pathways (Barnhart et al., 2004; Kreuz et al., 2004). Notably, caspase-8 acts through its scaffolding function to drive cytokines production in various cancer cell lines upon CD95L stimulation (Henry and Martin, 2017). Production of pro-inflammatory chemokines in dying cells results in the recruitment of monocytes and neutrophils that engulf the dying cells expressing the “find me” signal (Cullen et al., 2013). How CD95L triggers these apoptotic and non-apoptotic signaling pathways and their respective biologic functions remain to be better understood.

### CD95L

CD95 ligand also known as CD95L (FasL, TNFSF6 or CD178) is a type II transmembrane protein with a long cytoplasmic domain, a transmembrane (TM) domain, a stalk region, a TNF homology domain (THD) that mediates homotrimerization and a C-terminal region involved in CD95 binding (Figure 1C). The TM CD95L (membrane-CD95L or m-CD95L) can be cleaved in its stalk region by several matrix metalloproteases (MMPs) including MMP3, MMP7, MMP9, a disintegrin and metalloprotease-domain-containing protein (ADAM)-10 (Guegan and Legembre, 2018). The resulting soluble form of CD95L (s-CD95L) is a homotrimer (Tanaka et al., 1998) whose binding to CD95 fails to induce cell death (Suda et al., 1997; Schneider et al., 1998). Although the pathophysiological roles of s-CD95L remain to be elucidated, it accumulates in the bloodstream of patients suffering from a variety of diseases, including certain cancers such as NK cell lymphomas (Tanaka et al., 1996), ovarian cancers (De La Motte Rouge et al., 2019), and triple-negative breast cancer (TNBC) (Malleter et al., 2013). In TNBC women, high concentrations of s-CD95L are associated with the risk of relapse and metastatic dissemination (Malleter et al., 2013). s-CD95L levels are also elevated in inflammatory and autoimmune disorders such as systemic lupus erythematosus (SLE) (Tauzin et al., 2011; Poissonnier et al., 2016), rheumatoid arthritis (RA) (Hashimoto et al., 1998), and acute lung injury (ALI) (Herrero et al., 2011).

## CD95 Structure

**FIGURE 2:**
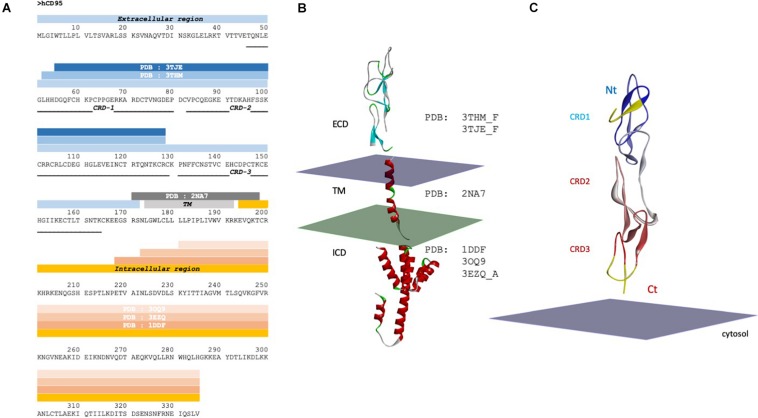
CD95 sequence and structure. **(A)** Sequence of CD95 with solved 3D structures and corresponding PDB ID code. Blue, gray and orange strips represent the extracellular domain, the transmembrane domain and the intracellular region of CD95, respectively. CRD, cysteine rich domain; TM, transmembrane; ICD, intracellular domain; ECD, extracellular domain. **(B)** Domains of a monomeric CD95 whose structure has been experimentally solved. The plasma membrane is symbolized by two parallel planes, with the outer leaflet in purple and the cytosolic couleur in green. Note that the orientation toward membrane is a hypothesis. **(C)** Structure of the extracellular domain of CD95. Crystal structure of CD95 ECD domain (PDB:3TJE), colored according to the sequence order (blue to red, from Nt to Ct extremities). The yellow structure (amino acid residues N31 to D55) represents the gap in the crystal structure, which has been completed using CD40 homology. Nt: Amino-terminal region; Ct: COOH-terminal region.

CD95 is detected homotrimerized independently of the presence of its ligand (Papoff et al., 1996; Siegel et al., 2000). Different domains in the death receptor seem to contribute to its aggregation, including the cytoplasmic DD (Ashkenazi and Dixit, 1998), TM and extracellular regions. Due to the TM nature, aggregation propensity and domain flexibility, the whole CD95 structure has not been solved yet. Nevertheless, 3D structures of some parts of the receptor have been deciphered by electron microscopy, X-ray crystallography or NMR spectroscopy (Figure 2A). Although CD95 structure has been extensively studied by these biophysical methods, the conformation of some important domains within the receptor, including a part of the pre-ligand assembly domain (PLAD) and the calcium-inducing domain (CID) (Figures 1B, 2A,B) are absent from these pictures, precluding a comprehensive understanding of the CD95-mediated cell signaling.

### Extracellular Region

The extracellular region of TNF receptors is characterized by the presence of cysteine-rich domains (CRDs), which contain six cysteine residues engaged in the formation of three disulfide bridges (Bodmer et al., 2002). The number of CRDs in a given receptor varies from one to four, and CD95 encompasses 3 CRDs (Figures 1B, 2C; Bodmer et al., 2002). The repeated and regular arrangement of CRDs confers an elongated shape to the receptors. In the absence of stimulation, CD95 is found at the plasma membrane as monomers or homodimers and homotrimers associated through their respective extracellular N-terminal PLAD, encompassing the amino acid residues 17–82 (or amino acid residues 1–66 according to the mature protein) (Papoff et al., 1999; Siegel et al., 2000). Accordingly, the elimination of PLAD in DD-mutated CD95 constructs abrogates their dominant-negative inhibitory effect, while expression of PLAD alone exerts a dominant negative action on the CD95-mediated apoptotic signal (Papoff et al., 1999; Siegel et al., 2000). More precisely, the minimal domain required for CD95 homotypic interaction contains amino acids 59–82 (43–66 without the peptide signal) (Edmond et al., 2012).

**FIGURE 3:**
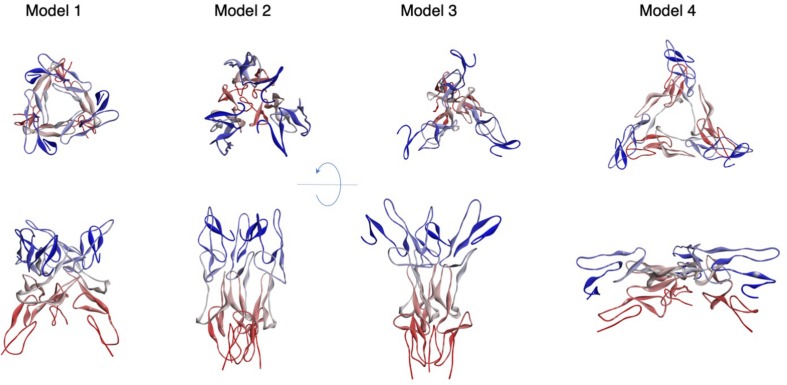
Models of trimeric CD95 ECD. Four models of the trimeric Apo CD95 ECD are depicted according to their decreasing docking score from left to right. Trimers were obtained by protomer docking, performed with SymmDock (Schneidman-Duhovny et al., 2005a). Ct ends are depicted in red, while the CD95 Nt region is in blue.

The structure of CD95 extracellular domain (ECD) has been determined after complexation with a Fab fragment of agonistic (i.e., apoptotic) anti-CD95 antibodies, bound to the CRDs 1 and 2 (Chodorge et al., 2012). The CD95/Fab complex is monomeric (Figure 2C), and although the antibody is an agonist, it shares only a short region with the CD95/CD95L interface, mainly the arginine at position 102 (based on the human CD95 precursor sequence with 16 amino acids subtracted to obtain the position on the mature sequence corresponding to R86, see Figure 1B). CD95 ECD exhibits a linear organization and its putative orientation to the membrane is predicted based on the solved Holo trimeric structure of other members of the TNFR family, including DcR3/CD95L (PDB:4MSV), DR5/TRAIL (1D0G), TNFR2/TNF (3ALQ), and DcR3/TL1A (3MI8). The PLAD residues consist of amino acids 17–82 (1–66 without the peptide signal) (Papoff et al., 1996; Siegel et al., 2000) and part of this region is missing from the X-ray data, i.e., amino acids arginine 17 (first amino acid following the peptide signal) to histidine 54 (Figure 1B). Also, the CD95 domains encompassing amino acid residues K148 to E156 and K164 to S170 are absent from X-ray and NMR analyses (Figure 2A). Threading approaches (Dunbrack, 2006) previously allowed us to build some plausible models of a completed ECD (including PLAD) in a trimeric organization (Levoin, 2017). However, in the present work focused on understanding of the trimeric assembly, we preferred not modeling a region whose structure is not strongly supported by experimental evidence. Therefore, to fill the two main gaps within the CD95 extracellular structure, we interrogated the PDB using PISA (Krissinel and Henrick, 2007) to find 3D homologs to the CD95 crystal structure (PDB : 3TJE). This method seems more appropriate than classical sequence-based screen, because sequence homology between DRs is rather low, and it is accepted that structure is more conserved than sequence (Murzin, 1998). Using this approach, we found that the structure of CD40 ECD (PDB : 3QD6) was close to that of CD95, with good geometric superposition and extended sequence solved (PISA Qscore = 0.55, with RMSD = 1.4 Å for 87 amino acids). Therefore, we completed the structure of CD95 protomer (residue N48 to E167, Figure 2B) using CD40 as a structural template. Afterward, each protomer served to assemble homotrimers through protein-protein docking, using SymmDock software (Schneidman-Duhovny et al., 2005b). We imposed a symmetric nature of the complex as a constraint, and the predicted four best solutions are presented Figure 3. These associations can be described as close Nt/remote Ct (model 1), close Nt/close Ct (model 2), remote Nt/close Ct (model 3), and remote Nt/remote Ct (model 4). The second solution is definitely the most compatible with biochemical data, because PLAD is known to be necessary and sufficient for receptor aggregation (Papoff et al., 1996; Siegel et al., 2000), and model 2 is the only one that orientates the amino terminal region in a way that can draw a large interface between PLADs. According to this computer-driven model, the interface between protomers occurred mostly between amino acid residues N48 to L52, K61 to P65 in CRD1, E114 to N118 in CRD2, R128 to V139 and C146 to E167 in CRD3. A noticeable structural feature of all trimer models is that protomers are tilted to the membrane, with an angle of about 45° for model 1 to 9° for model 2.

It is noteworthy that the homotypic PLAD affinity is rather low, almost in the mM range (Cao et al., 2011) suggesting that other domains in CD95 could exert a complementary role for the receptor homotrimerization, in agreement with the proposed trimeric model.

### Transmembrane Domain (TM)

Recent studies highlight that for several death receptors, the TM domain is involved in their aggregation. For example, DR5 TM helix promotes the assembly of high-order complexes responsible for cell death induction, independently of the ectodomain. Nuclear magnetic resonance (NMR) analysis of this TM region in bicelles shows different trimerization and dimerization interfaces responsible for a supramolecular dimer-trimer network (Pan et al., 2019). Surprisingly, elimination of the DR5 ECD triggers cell death in a TM-dependent and ligand (TRAIL)-independent manner, suggesting that the extracellular region of DR5 exerts an inhibitory action on the receptor activation; TRAIL binding overcoming this auto-inhibitory process (Pan et al., 2019).

**FIGURE 4:**
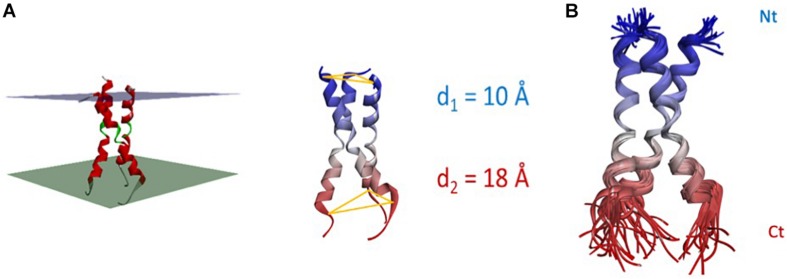
Structure and flexibility of CD95 TM. **(A)** Left panel: NMR-based structure of the trimeric TM helices according to PDB: 2NA7. The helix bundle is virtually inserted in a membrane, whose thickness is a hypothesis. Right panel: the Nt interdistance is less wide than its Ct counterpart (d1 = 9.7 ± 0.5 Å vs. d2 = 18.2 ± 2.6 Å between L174 or V195 Cα, for the 15 NMR structures). **(B)** Superposition of the 15 NMR structures, showing that the core of the bundle is quite rigid, while both ends are more flexible.

The CD95 TM domains have also been investigated by NMR in lipid/detergent bicelles (Fu et al., 2016) and these structures are found associated as stable trimers (Figure 4A). While the ends of the three helices display a certain flexibility, their core was more rigid (Figure 4B). The amino terminal portions of the helices (extracellular side) are closer and less flexible than their C-terminal counterparts (mean d1 = 9.7 ± 0.5 Å vs. d2 = 18.2 ± 2.6 Å, respectively, between L174 or V195 Cα of each protomer, for the 15 NMR structures). Unlike DR5, a proline motif is present in CD95 TM and in many members of the TNFR superfamily, including TNFR1, DR3, DR4, and CD40. This proline-rich sequence (P183 and P185 residues in the human sequence) within the CD95 TM helix favors packing of CD95 protomers through van der Waals interactions (Fu et al., 2016).

Transmembrane mutants affect the CD95L-mediated cell death program to a lesser extent than DD mutants (Fu et al., 2016). Indeed, co-expression of wild type and TM mutants does not disturb the formation of CD95 homotrimer in the absence of CD95L, indicating that the TM region of CD95 does not participate in its pre-ligand association (Fu et al., 2016). Nonetheless, CD95 TM domain probably stabilizes CD95 aggregation and/or conformation in the presence of CD95L because mutants within this domain impinge on the induction of apoptosis in cells exposed to CD95L (Fu et al., 2016).

Super-resolution microscopy data points out that monomers, dimers, and trimers of receptors co-exist on the plasma membrane before ligand binding, supporting that CD95 stoichiometry results from a dynamic equilibrium among oligomeric states (Fu et al., 2016), which could differ according to the expression level of the receptor itself and other factors that remain to be identified. Interestingly, somatic mutations exist in the human CD95 TM domain associated with malignancy, such as P183L associated with lymphoma or C178R mutation with squamous cell carcinoma (Tauzin et al., 2012) and these mutations abrogate the trimerization of TM domains in bicelles (Fu et al., 2016). Because TM mutants disrupting the CD95 homotrimerization impede the CD95L-induced apoptotic program, we can envision that this stoichiometry corresponds to its minimal arrangement required for induction of cell signaling.

### Intracellular Domain (ICD)

Like other death receptors, CD95 does not possess any intrinsic enzymatic activity and thereby initiates signaling cascades by recruiting proteins through protein-protein interactions (PPIs) in a dynamic manner. Most of the intracellular domain (ICD) is constituted by a DD, a scaffolding unit recruiting FADD through homotypic interactions. FADD in turn is a hub that binds caspase-8 and c-FLIP (Ferrao and Wu, 2012), and this complex cooperatively activates the apoptotic program (Hughes et al., 2016).

**FIGURE 5:**
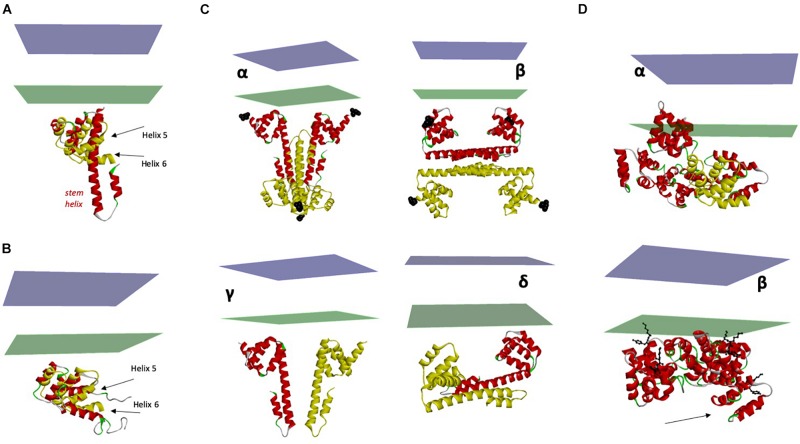
Structure and flexibility of CD95 ICD. **(A)** Superimposition of the two crystal structures of CD95 death domain (PDB:3EZQ in red and PDB:3OQ9 in yellow). In addition to the displacement of its juxtamembrane region, note the transformation of the α helices 5 and 6 within the death domain into a long stem helix. **(B)** Superimposition of Holo and Apo structures of the CD95 death domain (PDB:3OQ9 in yellow and PDB:1DDF in red). Note that there is still a conformational rearrangement of helices 5 and 6, but with a limited amplitude. **(C)** Different X-ray structures of ICD and their orientation toward the plasma membrane. Panels *α* to *δ*: proposed orientations of the tetrameric crystal structure of CD95:FADD complex (only CD95 is depicted). The N-terminal region of the death domain starting at N223 is the closest residue to TM and is labeled with black spheres. Chains in red seem correctly oriented regarding the plasma membrane, but the orientation of chains in yellow renders the position of the tetramer improbable. Panels γ to δ: only the closest dimers to the membrane are considered. Drawing in γ represents the most probable orientation toward the membrane. **(D)** Orientation of the pentameric CD95-DD, taking as reference the protomer showed in Figure 2B. Note that using this model, one DD is inserted into the plasma membrane. β. Optimized orientation of the pentameric CD95-DD regarding the position of the amino terminal residues K231 and Y232 (black balls and sticks) to the plasma membrane. Note the asymmetry of the structure, particularly for chain **(A)** (arrow).

The 3D structure of CD95 DD has been solved in complex with FADD by different teams (Figures 5A,B; Scott et al., 2009; Wang et al., 2010). Due to the different experimental conditions used in these studies, including low pH/high salt concentration vs. neutral pH and low salt concentration, and different methods (i.e., X-ray and NMR), the CD95 structures obtained are not completely superimposable (Figure 5A) and probably represent different conformational states of the domain. The X-ray structure of CD95 performed at pH 4 (Scott et al., 2009) reveals a dramatic shift in the carboxy-terminal region of the DD encompassing helices 5 and 6, resulting in the opening of the globular structure to render amino acids of the interface accessible to the FADD DD. This modification of the DD conformation was not detected in other X-ray studies of the CD95/FADD complex (Wang et al., 2010) or NMR analyses of CD95 alone (Huang et al., 1996) or combined with FADD (Esposito et al., 2010). Interestingly, mutations of residues within DD, which favor the opening of helix 6, enhance CD95-induced cell apoptosis, presumably because of an improved DISK formation. Unexpectedly, Driscoll’s team showed that shifting pH from 6.2 to 4 causes the loss of CD95/FADD interaction (Esposito et al., 2010) weakening the conclusions drawn at low pH or suggesting that acidic conditions could affect the way CD95 implements cell signaling (Monet et al., 2016).

The first crystallized CD95-DD/FADD complex showed a tetrameric arrangement (4:4) mostly mediated by CD95 domains (Figure 5C; Scott et al., 2009). However, the predicted orientation toward the membrane renders this model hardly compatible with the full assembly of the receptors. Indeed, Figure 5Cα illustrates that two chains of the tetramer are too far from the membrane. An alternative perpendicular orientation (Figure 5Cβ) seems also improbable for the same reason. Therefore, we suppose that this assembly results from crystal packing, and that a relevant biologic dimer is close to Figure 5Cγ. The second crystal structure of CD95 DD showed an asymmetric oligomeric complex composed of 5 CD95 DDs and 5 FADDs (Figure 5D; Wang et al., 2010). This latter study revealed that half of the residues involved in CD95/CD95, CD95/FADD or FADD/FADD interfaces are positively or negatively charged, suggesting again a sensitivity to salts or pH for the formation of the aggregated complex and thereby signal induction. Although the structure of this complex matches with the data obtained using electron microscopy, it remains questionable because rebuilt from the supposed orientation shown in Figure 5A, the structure is asymmetric, and one DD penetrates the membrane (Figure 5Dα). Optimization of the pentameric complex shows again a questionable asymmetry (Figure 5Dβ), even if the juxtamembrane region that we designated CID for Calcium-Inducing Domain (Figure 1B) is long and flexible enough to accommodate such a variability.

Calcium-inducing domain encompasses a 36 amino-acids sequence (amino acids K191 to D226), which is predicted to be disordered, explaining why it has never been solved by structural studies. Molecular modeling can, however, illuminate this structure at a single molecule level, showing that CID presented sparsely and transiently folded small α helix (Poissonnier et al., 2016). The role of this peptide in the DD conformation and orientation to the plasma membrane and thereby in the recruitment of FADD is difficult to predict.

While the DD (amino acid residues 210–303) is involved in cell death, the biological roles of the last 15 residues of CD95 (amino acids 303–319) remain largely unknown. The protein tyrosine phosphatase FAP-1 (Sato et al., 1995) or Dlg1 (Gagnoux-Palacios et al., 2018) can interact with this carboxy-terminal region and inhibit cell death, through unknown molecular mechanisms.

In conclusion, 3D structures of CD95 combined with biochemical and cellular data suggest the existence of different conformations for CD95-DD but their roles in the recruitment of FADD or other partners and the implementation of cell signaling remain to be understood.

### Reconstitution of a Whole CD95 ECD/TM Structure

#### Apo CD95

**FIGURE 6:**
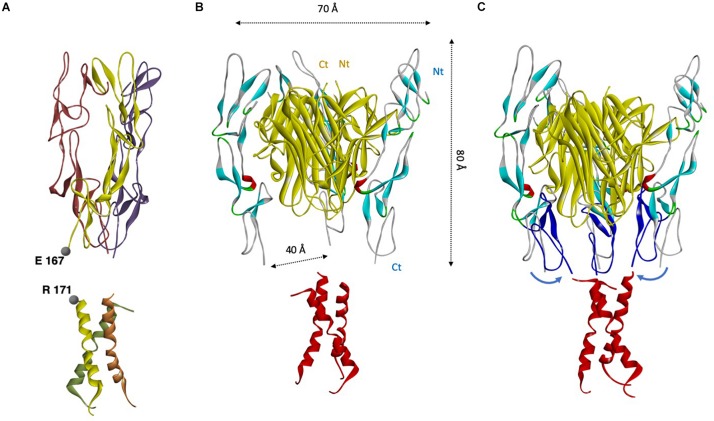
CD95/CD95L complex and its association to the homotrimeric CD95 TM. **(A)** Manual alignment of trimeric Apo CD95 ECD model and experimental trimeric TM. Only residues E_168_GS are missing, and ECD E167 and TM R171 appeared close. **(B)** CD95-ECD was rebuilt using CD40 structure as a template. Next, this protomer was geometrically superimposed to the DcR3 homotrimer structure interacting with homotrimeric CD95L (yellow) with Maestro, Schrödinger Inc. (optimization of the α carbons superposition, with a final RMSD of 7.5 Å between DcR3 and CD95). CD95 TM is depicted in red. **(C)** Unlike the Apo CD95-ECD, the Holo Ct ends of CD95-ECD are too remote (each Ct end is distant from 40 Å) to be connected to the Nt ends of CD95-TM (in red) by the only 3 missing amino acids. The most probable rearrangement following ligand binding is a rotation/rocking of the whole CRD3 depicted in navy blue around N132 (the resulting modeled position is in navy blue ribbons, marked by blue arrows).

Superposition of the trimeric model 2 shown in Figure 3 to the experimental TM bundle showed a near perfect alignment (Figure 6A). The only 3 residues lacking in the ECD (i.e., E_168_GS) near the outer leaflet of the plasma membrane could form a short loop with a flexible glycine. This loop can easily fill the gap between ECD and TM, supporting our trimeric model 2. Indeed, the estimated distance between α carbon of each protomer of CD95 ECD at position E167 corresponds to 51 Å for model 1, 18 Å for model 2, 14 Å for model 3, and 64 Å for model 4, while trimeric NMR-based TM showed an average distance of 21 Å between α carbon at position R171 (Fu et al., 2016).

#### Holo CD95

The structure of the CD95L complexed with the decoy receptor DcR3 in a trimeric complex has been solved (Liu et al., 2016). Based on structural similarities, we superimposed our previously rebuilt CD95 ECD to DcR3 receptor, resulting in a trimeric CD95L/CD95 complex (Figure 6B). The homotrimeric Apo CD95 structure exhibits a packed conformation (Figure 6A), so a large opening of this quaternary structure is necessary to allow the insertion of the CD95L homotrimer (Figure 6B). In this Holo conformation, the structural organization of the CD95L/CD95 trimer reveals that the missing three amino acids of CD95 ECD cannot fill anymore the gap between ECD and TM, with a distance between α carbon of each ECD CD95 at position E167 of 39 Å (Figure 6B). This observation raises two hypotheses: either the distance of the TM bundle changes between Apo and Holo CD95 trimers, or CD95 CRD3 is very flexible and naturally pivots under CD95L to cover partially its bottom side, probably around the hinge formed by N132 (Figure 6C). The second scenario seems the most plausible, because, first, TM domain has to be trimeric to implement the apoptotic signaling pathway in the presence of CD95L (Fu et al., 2016), and second, the lack of electron density in the crystal structure of CD95 CRD3 suggests a flexible domain. Moreover, the slope of the CD95 ECD protomers inside the trimer is reminiscent of an inverted iris-like mechanism observed for certain channels and transporters (Yoder et al., 2018; McCarthy et al., 2019), in which the inducer engenders a small conformation change, echoed into a huge amplitude modification at the opposite end of the structure. Marchesi et al. (2018) recently theorized the mechanical lever effect of the iris-like motion, and concluded that this mechanism reduces by 3 the force required to open channels. If this iris-like mechanism permits an amplification of motion from ECD to TM in response to small extracellular ligands (i.e., cyclic nucleotide and ATP), an inverted physical principle might here switch an important extracellular movement (i.e., insertion of a large ligand) into a minimal TM perturbation. Based on this mechanism, the trimeric TM would not need to dissociate when CD95 ECD widely opens to accept the homotrimeric CD95L. Moreover, a small motion in the juxtamembrane hinge region (for example, E_168_GS) should counterbalance the large shift of the PLAD domain (Figure 6C). In agreement with a movement of the CD95 juxtamembrane domain, we estimated in our model a distance of 7 Å between the CD95 residue S137 and its partner on CD95L (P206), which was shown critical for the interaction (Schneider et al., 1997). Because this distance is too important for an implication of P206 in CD95/CD95L interaction, the receptor requires to approach the ligand, either through CRD3 flexibility or by a rotation/rocking of the receptor protomer following the iris-like hypothesis. These conformational rearrangements will require further investigations using normal mode analysis (Wako and Endo, 2017) and molecular dynamics (Arroyo-Manez et al., 2011; Wang et al., 2018).

## External Factors Mediated Stoichiometry

### CD95 Post-translational Modifications (PTMs)

CD95 can be glycosylated and different reports indicate that sialylation of asparagines 118 and 136 (corresponding to N102 and N120 in the human mature CD95 protein), improves the induction of the cell death program (Peter et al., 1995; Keppler et al., 1999). However, more recent data challenged the involvement of these glycosylations in the induction of cell death (Shatnyeva et al., 2011). Because the elimination of these glycosylations do not affect the stability or the plasma membrane expression of CD95 (Shatnyeva et al., 2011), it could be interesting in the future to explore the effect of these PTMs on the induction of the CD95-mediated non-apoptotic signaling pathways.

Several other PTMs affect the extent of oligomerization of CD95 prior to or following its interaction with CD95L. These include S-palmitoylation of the juxtamembrane cysteine at position 199 (Chakrabandhu et al., 2007; Feig et al., 2007) and S-nitrosylations on both C199 and C304 (Leon-Bollotte et al., 2011). S-glutathionylation of CD95 at cysteine 294 (mouse amino acid sequence) promotes its aggregation and subsequent caspase activation and apoptosis (Anathy et al., 2009). Glutaredoxins (Grxs) reverse this process. Therefore, reactive oxygen species (ROS) can enhance CD95-mediated caspase-8 activation, which in turn cleaves and inactivates Grx1, generating a positive feedback loop sealing the cell fate. Also, CD95 S-glutathionylation promotes its distribution into lipid rafts and its avidity for CD95L (Anathy et al., 2009). These results highlight that CD95 aggregation and signaling can be modulated by a redox-based mechanism.

Phosphorylation of CD95 on different serine/threonine and tyrosine (Y232 and Y291) within its intracellular region can modulate its signaling pathways (Chakrabandhu and Hueber, 2016). The replacement of Y291 by phenylalanine prevents recruitment of the AP-2 adaptor complex and the subsequent clathrin-mediated CD95 internalization but does not affect FADD binding and cell death induction (Lee et al., 2006). Interestingly, although this Y291 mutation inhibits the induction of the apoptotic signaling pathway (Lee et al., 2006), it fails to alter the induction of non-apoptotic signals such as NF-KB and MAPK (Lee et al., 2006) suggesting that similarly to TNF-R1 signaling (Micheau and Tschopp, 2003), apoptotic and non-apoptotic machinery are assembled within different sub-cellular localizations.

In addition to receptor tyrosine kinases (RTKs) described below (see paragraph III-2), src-family kinases (SFKs) can phosphorylate tyrosines in CD95 leading to the inhibition of the apoptotic program and these phosphorylation marks might serve as poor prognostic markers in several types of cancer, including breast, ovarian, and colon cancers (Chakrabandhu et al., 2016). Of note, this Y291 phosphorylation can also recruit some phosphatases including SHP-1 and SHP-2 and SH2-containing inositol phosphatase (SHIP), whose activities counteract the granulocyte–macrophage colony-stimulating factor (GM-CSF)-mediated pro-survival signal in neutrophils (Daigle et al., 2002). In conclusion, phosphorylation of Y291 within DD of CD95 might participate in the inhibition of the CD95-mediated apoptotic pathway and at least in certain cells including neutrophils, might terminate the cytokine-mediated pro-survival signaling pathways rendering difficult to predict the role of this PTM in the cell fate.

### CD95 ECD Partners

The tyrosine-protein kinase c-Met, also known as hepatocyte growth factor receptor (HGFR), can be associated with CD95, *via* a YLGA amino-acids sequence located in the N-terminal region of the c-Met α-chain (Wang et al., 2002), and CD95L also bears a ^244^YLGA^247^ sequence. Nonetheless, the observed competition between c-Met and CD95L for CD95 interaction raises some questions because the YLGA-containing c-Met sequence (i) competes with CD95L for CD95 binding, despite the fact that the CD95/CD95L interface involves amino acid residues different from the CD95L YLGA sequence (Schneider et al., 1997) and (ii) seems to disrupt CD95 oligomerization even if the CD95/CD95 aggregation requires CRD1 (PLAD) and TM domains different from the CRD2 and CRD3 regions involved in CD95/CD95L interface. Therefore, it remains to better understand how c-Met and CD95 interact to elucidate how this receptor affects the CD95 signaling pathway.

Of note, an additional RTK, namely epidermal growth factor receptor (EGFR) has been linked to the modulation of the CD95-mediated signaling pathway. Accordingly, Haussinger’s team reported that the hydrophobic bile salts can trigger cell death in hepatocytes through activation of EGFR, which induces CD95 tyrosine phosphorylation and implementation of cell death (Reinehr et al., 2003a, b). By contrast, other groups established that the EGFR-induced MAPK pathway counteracts CD95-mediated apoptosis in hepatocyte cells exposed to bile salts (Qiao et al., 2001) and this RTK also inhibits the CD95-mediated apoptotic signaling pathway in glioma cells (Steinbach et al., 2002) rendering difficult to conclude on the role of EGFR in the modulation of the CD95-mediated cell death program. On the other hand, the presence of EGFR exerts a pivotal role in the induction of the CD95-mediated non-apoptotic signaling pathways. In the presence of CD95L, CD95 recruits EGFR to implement the PI3K signaling pathway in TNBC cells (Malleter et al., 2013) or the MAPK pathway (i.e., extracellular signal-regulated kinase) in hepatic stellate cells (HSCs) (Reinehr et al., 2008) and thereby promotes cell migration or proliferation, respectively.

Interestingly, a recent study highlighted the role of CD95 in sensing the cell survival of epithelial cells and thereby the maintain of tissue integrity (Gagnoux-Palacios et al., 2018). In adherens junction, the proximity of E-cadherin and α-catenin to CD95 favors the recruitment of Dlg1 to the C-terminal region of CD95 (Gagnoux-Palacios et al., 2018). Dlg1impinges on the DISK formation in cells exposed to m-CD95L and the loss of adherens junction will favor the release of this anti-apoptotic factor to promote cell death, a mechanism that could prevent metastatic dissemination of pre-tumor cells. Another method for adhesion molecules to control cell death has been also established for ICAM-2 (Perez et al., 2002). ICAM-2 over-expression or its interaction with leukocyte function-antigen-1 (LFA-1) induces ezrin phosphorylation by src tyrosine kinase and PI3K/AKT activation (Perez et al., 2002), which in turn impairs the induction of the CD95-mediated apoptotic program in leukocytes. This study points out that the PI3K activation by adhesion molecules can protect cells from apoptotic signal induced by death receptors.

### Ion-Driven CD95 Stoichiometry

As aforementioned, upon addition of CD95L, CD95 undergoes conformational modification of its DD, inducing a shift of helix 6 and fusion with helix 5, promoting both oligomerization of the receptor and recruitment of the adaptor protein FADD (Scott et al., 2009). However, the idea of an elongated C-terminal α-helix favoring the *cis-*dimerization of CD95-DD in the acidic conditions (pH 4) was challenged by Driscoll and colleagues (Esposito et al., 2010) who did not observe the fusion of the last two helices at a more neutral pH (pH 6.2). These findings raise the question of whether a local decrease in intracellular pH might affect the CD95 conformation by promoting the opening of the CD95-DD and eventually by contributing to the formation of a complex that elicits a sequence of events distinct from what occurs at physiologic pH. Accordingly, we recently observed that CD95 activates the Na^+^/H^+^ exchanger 1 (NHE1) in the presence of s-CD95L (Monet et al., 2016). NHE1 catalyzes an electroneutral exchange of extracellular Na^+^ for intracellular H^+^, and its activity is necessary for cell migration (Putney et al., 2002; Frantz et al., 2007). While the presence of s-CD95L activates NHE1, no such modulation is observed in cells stimulated with a cytotoxic, multi-aggregated CD95L, suggesting that an acidic pH may surround the intracellular region of CD95 in cells stimulated with cytotoxic CD95L as compared to that in cells exposed to s-CD95L (Monet et al., 2016). This observation might explain how a drop of pH close to CD95 could promote a receptor conformation recruiting FADD and thereby, unleash the apoptotic signaling pathway.

By contrast, NHE1 activation by CD95 (Monet et al., 2016) alkalinizes the intracellular region and could prevent modification of DD helix 5 and 6 fusion (Scott et al., 2009). Of note, acidification can affect the protein conformation through the modulation of histidines as demonstrated for the phosphoinositide binding of cofilin, which is pH-dependent and decreases at high pH (Frantz et al., 2008). Interestingly, the intracellular region of CD95 encompasses four histidines and two of them (H282 and H285, i.e., H266 and H269 in the mature protein) are localized upstream helix 5 (Tauzin et al., 2012).

### Lipid-Driven CD95 Stoichiometry

CD95 aggregation relies on the plasma membrane composition in lipids. Indeed, CD95 aggregation is slower in 1,2-dipalmitoyl-sn-glycero-3-phosphocholine (DPPC) than in 1,2-dioleoyl-sn-glycero-3-phosphocholine (DOPC), thereby the apoptotic program is faster in the latter lipid environment (Gulculer Balta et al., 2019). CD95 engagement triggers the accumulation of ceramide in a caspase-8-dependent manner, which in turn contributes to its aggregation and thereby favors the induction of cell death (Grassme et al., 2003). The initial stage of the CD95 response could be described as a two-step process, first requiring a certain degree of CD95 aggregation to secondarily promoting a caspase-8-driven intracellular signaling pathway that results in the aggregation and distribution of unstimulated CD95 into lipid rafts (Siegmund et al., 2017) which seems to favor the apoptotic response (Gajate et al., 2004). S-palmitoylation of CD95 (Chakrabandhu et al., 2007; Feig et al., 2007) appears to promote CD95 redistribution into lipid rafts (Muppidi and Siegel, 2004; Chakrabandhu et al., 2007).

### Ligand-Mediated CD95 Stoichiometry

#### Two Ligands

Contrary to its receptor, CD95L is a type II transmembrane protein whose N-term extremity is in the cytoplasm (Figure 1C). The membrane-bound native CD95L (m-CD95L) can be processed by several proteases, including MMP3, MMP7, MMP9, and ADAM-10 (Tauzin et al., 2012), to release a soluble form of the ligand (s-CD95L) (Figure 1C). m-CD95L is responsible for the DISK assembly, whereas s-CD95L can trigger the formation of a different complex designated MISC (Malleter et al., 2013; Poissonnier et al., 2016). While most of the studies on s-CD95L report that this ligand possesses an homotrimeric stoichiometry, its membrane-bound counterpart shows a higher degree of aggregation such as large synapse of CD95L are observed between CD95L-expressing T-cells or NK cells and their cellular targets. Human CD95L self-association domain spans between amino acid residues 137–183 (Voss et al., 2008) and the last three amino acids are important for CD95 interaction (Figure 1C; Orlinick et al., 1997). CD95L ectodomain contains three putative sites for N-linked glycosylation (N184, N250, and N260) and a lack of glycosylation alters the expression level of this ligand, probably by acting on its stability and/or intracellular trafficking (Schneider et al., 1997; Voss et al., 2008). Although CD95L/CD95 structure and surface plasmon resonance analyses reveal that CD95L glycosylation is not interfering with CD95 binding, the glycosylated ligand triggers a stronger cell death signal as compared to its sugar-free counterpart (Liu et al., 2016). While this difference of function has been associated with the fact that CD95L glycosylation might reduce the magnitude of its aggregation level (Liu et al., 2016), other studies showed no effect on the induction of cell death signal (Orlinick et al., 1997; Schneider et al., 1997; Voss et al., 2008), rendering difficult to conclude on the exact biological role played by the N-glycosylation of CD95L. Although O-glycosylation for DR5 (Wagner et al., 2007) and N-glycosylation for DR4 do not alter the intensity of their interaction with TRAIL (Dufour et al., 2017), these PTMs enhance the apoptotic signal through molecular mechanisms that remain to be elucidated (Micheau, 2018). A possible explanation could come from galectins, which are small proteins capable to bind to the β-galactoside sugars present in the extracellular region of TNF receptors family members. Of note, different galectins can bind and aggregate DR4, DR5, and CD95 and thereby, stimulate or inhibit cell death (Micheau, 2018) suggesting the subtle role played by glycosylation in the implementation of cell signaling by death receptors.

#### CD95 Ligands and Aggregation

The role of CD95L in the extend of CD95 aggregation is not clearly understood. Although most studies report that the metalloprotease-cleaved CD95L engenders homotrimer unable to induce cell death (Tanaka et al., 1996, 1998; Tauzin et al., 2011; Suda et al., 1997; Schneider et al., 1998) but instead triggers pro-inflammatory signaling pathways, in certain pathologies, a soluble CD95L is accumulated and reaches an aggregation level of CD95 allowing the implementation of the cell death program (Bajou et al., 2008; Herrero et al., 2011). Moreover, although the homotrimeric s-CD95L does not induce cell death, a recombinant and hexameric form does (Holler et al., 2003), supporting that the extent to which CD95L is multimerized is a pivotal step in determining whether non-apoptotic signaling or cell death is induced. Notably, some pathophysiological conditions could favor s-CD95L oligomerization, thereby promoting its cytotoxic activity. CD95L in the bronchoalveolar lavage (BAL) fluid of patients suffering from acute respiratory distress syndrome (ARDS) undergoes oxidation at methionines 224 and 225, promoting its aggregation and thereby rendering it cytotoxic (Herrero et al., 2011). In addition, in ARDS BAL fluid, another methionine oxidation occurs at position 121 within CD95 and prevents its cleavage by MMP7 which can explain why this cytotoxic ligand retains its stalk region (Herrero et al., 2011). Nonetheless, whether this corresponds to an alternatively cleaved form of s-CD95L with higher-level stoichiometry or a full-length exosome-bound m-CD95L remains to be elucidated.

The stoichiometry of CD95L can also be increased by external elements including fibronectin in the extracellular matrix (Aoki et al., 2001), rendering the inactive molecule apoptotic and raising the question of which domain within the soluble ligand interacts with fibronectin.

The different responses obtained with soluble and membrane-bound CD95L are a common feature among the TNF superfamily. For instance, while soluble TNF binds efficiently both TNFR1 and TNFR2, it stimulates TNFR1 signaling and induces cell death, but fails to trigger any response with TNFR2 (Grell et al., 1995). More importantly, the artificial oligomerization of soluble ligands restores the implementation of a classical response (Wajant, 2015) indicating that the ligand stoichiometry modulates the cell signaling in this superfamily. However, the difference between soluble and membrane-bound ligand signaling can be not so tremendous that what is observed for TNR2 or CD95. For instance, membrane TWEAK (TNF-like weak inducer of apoptosis) induces both alternative and classical NFκB pathways while soluble TWEAK only triggers the classical NFκB pathway (Wajant, 2015).

#### Agonistic Antibodies

An interesting study using a set of agonistic anti-CD95 antibodies revealed an inverse correlation between antibody affinity and cell death (Chodorge et al., 2012). A structure–function analysis disclosed that dissociation rate (Koff) of anti-CD95 antibodies is crucial for receptor activation because beyond affinity, dissociation of one antibody arm allows antibodies to bring together more CD95 monomers, forming a receptor cluster required to trigger cell death (Chodorge et al., 2012). These observations strengthen that the level of CD95 aggregation is important to induce the cell death process. However, the role of aggregation in the induction of non-apoptotic signaling pathways has not been investigated in this study and could be interesting to address.

## Discussion

Unsurprisingly, initial therapeutic solutions involving CD95 focused on the apoptotic pathway. Most of research efforts have dealt on deciphering the molecular basis of apoptosis induction by CD95 and considering the biological functions of CD95 in light of this role. Although non-apoptotic functions of CD95 (Alderson et al., 1993) have been reported soon after CD95 cloning (Itoh et al., 1991; Alderson et al., 1993), these have been largely neglected over the years. As a consequence, no CD95 agonists have become a standard of care in inflammatory disorders or cancers. It is now clear that CD95 can contribute to multiple biological functions, including inflammation and tumorigenesis through the induction of non-apoptotic signaling pathways. Accordingly, a CD95 decoy receptor blocking both the apoptotic and non-apoptotic signaling pathways, Asunercept (APG101), has nevertheless entered clinical trials for glioma and myelodysplastic syndrome (Wick et al., 2014; Boch et al., 2018).

Overall, the evidence that homotrimeric ligand can activate certain receptor-associated signaling pathways favors the concept of a two-step model of TNFRSF activation. In a first step, there is ligand induced formation of homotrimeric TNFSF/TNFRSF complex, triggering some signaling pathways (mainly non-apoptotic signaling pathways). In a second step, there is multimerization of the homotrimeric complex through different mechanisms including oligomerization, transactivation, plasma membrane or microdomain redistribution/exclusion inducing different signaling pathways. Each of these steps constitute possible targets for therapeutic agents and should be scrutinized in future studies.

## Author Contributions

NL and PL designed the experiments (computer modeling) and wrote the manuscript. MJ wrote the manuscript.

## Conflict of Interest

The authors declare that the research was conducted in the absence of any commercial or financial relationships that could be construed as a potential conflict of interest. The handling Editor declared a past co-authorship with one of the authors PL.

## References

[B1] Alderson M. R. Armitage R. J. Maraskovsky E. Tough T. W. Roux E. Schooley K. (1993). Fas transduces activation signals in normal human T lymphocytes. *J. Exp. Med.* 178 2231–2235. 10.1084/jem.178.6.2231 7504062 PMC2191272

[B2] Anathy V. Aesif S. W. Guala A. S. Havermans M. Reynaert N. L. Ho Y. S. (2009). Redox amplification of apoptosis by caspase-dependent cleavage of glutaredoxin 1 and S-glutathionylation of Fas. *J. Cell Biol.* 184 241–252. 10.1083/jcb.200807019 19171757 PMC2654302

[B3] Aoki K. Kurooka M. Chen J. J. Petryniak J. Nabel E. G. Nabel G. J. (2001). Extracellular matrix interacts with soluble CD95L: retention and enhancement of cytotoxicity. *Nat. Immunol.* 2 333–337. 10.1038/86336 11276204

[B4] Arroyo-Manez P. Bikiel D. E. Boechi L. Capece L. Di Lella S. Estrin D. A. (2011). Protein dynamics and ligand migration interplay as studied by computer simulation. *Biochim. Biophys. Acta* 1814 1054–1064. 10.1016/j.bbapap.2010.08.005 20797453

[B5] Ashkenazi A. Dixit V. M. (1998). Death receptors: signaling and modulation. *Science* 281 1305–1308. 10.1126/science.281.5381.1305 9721089

[B6] Bajou K. Peng H. Laug W. E. Maillard C. Noel A. Foidart J. M. (2008). Plasminogen activator inhibitor-1 protects endothelial cells from FasL-mediated apoptosis. *Cancer Cell* 14 324–334. 10.1016/j.ccr.2008.08.012 18835034 PMC2630529

[B7] Barnhart B. C. Legembre P. Pietras E. Bubici C. Franzoso G. Peter M. E. (2004). CD95 ligand induces motility and invasiveness of apoptosis-resistant tumor cells. *EMBO J.* 23 3175–3185. 10.1038/sj.emboj.7600325 15272306 PMC514938

[B8] Boch T. Luft T. Metzgeroth G. Mossner M. Jann J. C. Nowak D. (2018). Safety and efficacy of the CD95-ligand inhibitor asunercept in transfusion-dependent patients with low and intermediate risk MDS. *Leuk. Res.* 68 62–69. 10.1016/j.leukres.2018.03.007 29549809

[B9] Bodmer J. L. Schneider P. Tschopp J. (2002). The molecular architecture of the TNF superfamily. *Trends Biochem. Sci.* 27 19–26. 10.1016/s0968-0004(01)01995-8 11796220

[B10] Cao J. Meng F. Gao X. Dong H. Yao W. (2011). Expression and purification of a natural N-terminal pre-ligand assembly domain of tumor necrosis factor receptor 1 (TNFR1 PLAD) and preliminary activity determination. *Protein J.* 30 281–289. 10.1007/s10930-011-9330-4 21574063

[B11] Chakrabandhu K. Herincs Z. Huault S. Dost B. Peng L. Conchonaud F. (2007). Palmitoylation is required for efficient Fas cell death signaling. *EMBO J.* 26 209–220. 10.1038/sj.emboj.7601456 17159908 PMC1782379

[B12] Chakrabandhu K. Huault S. Durivault J. Lang K. Ta Ngoc L. Bole A. (2016). An evolution-guided analysis reveals a multi-signaling regulation of fas by tyrosine phosphorylation and its implication in human cancers. *PLoS Biol.* 14:e1002401. 10.1371/journal.pbio.1002401 26942442 PMC4778973

[B13] Chakrabandhu K. Hueber A. O. (2016). Fas versatile signaling and beyond: pivotal role of tyrosine phosphorylation in context-dependent signaling and diseases. *Front. Immunol.* 7:429. 10.3389/fimmu.2016.00429 27799932 PMC5066474

[B14] Chan F. K. Chun H. J. Zheng L. Siegel R. M. Bui K. L. Lenardo M. J. (2000). A domain in TNF receptors that mediates ligand-independent receptor assembly and signaling. *Science* 288 2351–2354. 10.1126/science.288.5475.2351 10875917

[B15] Chodorge M. Zuger S. Stirnimann C. Briand C. Jermutus L. Grutter M. G. (2012). A series of Fas receptor agonist antibodies that demonstrate an inverse correlation between affinity and potency. *Cell Death Differ.* 19 1187–1195. 10.1038/cdd.2011.208 22261618 PMC3374083

[B16] Cullen S. P. Henry C. M. Kearney C. J. Logue S. E. Feoktistova M. Tynan G. A. (2013). Fas/CD95-induced chemokines can serve as “find-me” signals for apoptotic cells. *Mol. Cell* 49 1034–1048. 10.1016/j.molcel.2013.01.025 23434371

[B17] Daigle I. Yousefi S. Colonna M. Green D. R. Simon H. U. (2002). Death receptors bind SHP-1 and block cytokine-induced anti-apoptotic signaling in neutrophils. *Nat. Med.* 8 61–67. 10.1038/nm0102-61 11786908

[B18] De La Motte Rouge T. Corne J. Cauchois A. Le Boulch M. Poupon C. Henno S. (2019). Serum CD95L level correlates with tumor immune infiltration and is a positive prognostic marker for advanced high-grade serous ovarian cancer. *Mol. Cancer Res.* 17 2537–2548. 10.1158/1541-7786.MCR-19-0449 31537619

[B19] Desbarats J. Birge R. B. Mimouni-Rongy M. Weinstein D. E. Palerme J. S. Newell M. K. (2003). Fas engagement induces neurite growth through ERK activation and p35 upregulation. *Nat. Cell Biol.* 5 118–125. 10.1038/ncb916 12545171

[B20] Desbarats J. Newell M. K. (2000). Fas engagement accelerates liver regeneration after partial hepatectomy. *Nat. Med.* 6 920–923. 10.1038/78688 10932231

[B21] Dostert C. Grusdat M. Letellier E. Brenner D. (2019). The TNF family of ligands and receptors: communication modules in the immune system and beyond. *Physiol. Rev.* 99 115–160. 10.1152/physrev.00045.2017 30354964

[B22] Dufour F. Rattier T. Shirley S. Picarda G. Constantinescu A. A. Morle A. (2017). N-glycosylation of mouse TRAIL-R and human TRAIL-R1 enhances TRAIL-induced death. *Cell Death Differ.* 24 500–510. 10.1038/cdd.2016.150 28186505 PMC5344210

[B23] Dunbrack R. L. Jr. (2006). Sequence comparison and protein structure prediction. *Curr. Opin. Struct. Biol.* 16 374–384. 10.1016/j.sbi.2006.05.006 16713709

[B24] Edmond V. Ghali B. Penna A. Taupin J. L. Daburon S. Moreau J. F. (2012). precise mapping of the CD95 pre-ligand assembly domain. *PLoS One* 7:e46236. 10.1371/journal.pone.0046236 23049989 PMC3457997

[B25] Esposito D. Sankar A. Morgner N. Robinson C. V. Rittinger K. Driscoll P. C. (2010). Solution NMR investigation of the CD95/FADD homotypic death domain complex suggests lack of engagement of the CD95 C terminus. *Structure* 18 1378–1390. 10.1016/j.str.2010.08.006 20947025

[B26] Feig C. Tchikov V. Schutze S. Peter M. E. (2007). Palmitoylation of CD95 facilitates formation of SDS-stable receptor aggregates that initiate apoptosis signaling. *EMBO J.* 26 221–231. 10.1038/sj.emboj.7601460 17159907 PMC1782382

[B27] Felsenstein J. (1989). Mathematics vs. Evolution: mathematical evolutionary theory. *Science* 246 941–942. 10.1126/science.246.4932.941 17812579

[B28] Ferrao R. Wu H. (2012). Helical assembly in the death domain (DD) superfamily. *Curr. Opin. Struct. Biol.* 22 241–247. 10.1016/j.sbi.2012.02.006 22429337 PMC3320699

[B29] Frantz C. Barreiro G. Dominguez L. Chen X. Eddy R. Condeelis J. (2008). Cofilin is a pH sensor for actin free barbed end formation: role of phosphoinositide binding. *J. Cell Biol.* 183 865–879. 10.1083/jcb.200804161 19029335 PMC2592832

[B30] Frantz C. Karydis A. Nalbant P. Hahn K. M. Barber D. L. (2007). Positive feedback between Cdc42 activity and H+ efflux by the Na-H exchanger NHE1 for polarity of migrating cells. *J. Cell Biol.* 179 403–410. 10.1083/jcb.200704169 17984318 PMC2064788

[B31] Fu Q. Fu T. M. Cruz A. C. Sengupta P. Thomas S. K. Wang S. (2016). Structural basis and functional role of intramembrane trimerization of the Fas/CD95 death receptor. *Mol. Cell* 61 602–613. 10.1016/j.molcel.2016.01.009 26853147 PMC4761300

[B32] Gagnoux-Palacios L. Awina H. Audebert S. Rossin A. Mondin M. Borgese F. (2018). Cell polarity and adherens junction formation inhibit epithelial Fas cell death receptor signaling. *J. Cell Biol.* 217 3839–3852. 10.1083/jcb.201805071 30242034 PMC6219722

[B33] Gajate C. Del Canto-Janez E. Acuna A. U. Amat-Guerri F. Geijo E. Santos-Beneit A. M. (2004). Intracellular triggering of Fas aggregation and recruitment of apoptotic molecules into Fas-enriched rafts in selective tumor cell apoptosis. *J. Exp. Med.* 200 353–365. 10.1084/jem.20040213 15289504 PMC2211978

[B34] Gao L. Gulculer G. S. Golbach L. Block H. Zarbock A. Martin-Villalba A. (2016). Endothelial cell-derived CD95 ligand serves as a chemokine in induction of neutrophil slow rolling and adhesion. *eLife* 5:e18542. 10.7554/eLife.18542 27763263 PMC5098908

[B35] Grassme H. Cremesti A. Kolesnick R. Gulbins E. (2003). Ceramide-mediated clustering is required for CD95-DISC formation. *Oncogene* 22 5457–5470. 10.1038/sj.onc.1206540 12934106

[B36] Grell M. Douni E. Wajant H. Lohden M. Clauss M. Maxeiner B. (1995). The transmembrane form of tumor necrosis factor is the prime activating ligand of the 80 kDa tumor necrosis factor receptor. *Cell* 83 793–802. 10.1016/0092-8674(95)90192-2 8521496

[B37] Guegan J. P. Legembre P. (2018). Nonapoptotic functions of Fas/CD95 in the immune response. *FEBS J.* 285 809–827. 10.1111/febs.14292 29032605

[B38] Gulculer Balta G. S. Monzel C. Kleber S. Beaudouin J. Balta E. Kaindl T. (2019). 3D Cellular architecture modulates tyrosine kinase activity, thereby switching CD95-mediated apoptosis to survival. *Cell Rep.* 29 2295–2306.e6. 10.1016/j.celrep.2019.10.054 31747602

[B39] Hashimoto H. Tanaka M. Suda T. Tomita T. Hayashida K. Takeuchi E. (1998). Soluble Fas ligand in the joints of patients with rheumatoid arthritis and osteoarthritis. *Arthritis Rheum.* 41 657–662. 10.1002/1529-0131(199804)41:4<657::aid-art12>3.0.co;2-n 9550474

[B40] Henry C. M. Martin S. J. (2017). Caspase-8 acts in a non-enzymatic role as a scaffold for assembly of a pro-inflammatory “FADDosome” complex upon TRAIL stimulation. *Mol. Cell* 65 715–729.e5. 10.1016/j.molcel.2017.01.022 28212752

[B41] Herrero R. Kajikawa O. Matute-Bello G. Wang Y. Hagimoto N. Mongovin S. (2011). The biological activity of FasL in human and mouse lungs is determined by the structure of its stalk region. *J. Clin. Invest.* 121 1174–1190. 10.1172/JCI43004 21285513 PMC3049393

[B42] Holler N. Tardivel A. Kovacsovics-Bankowski M. Hertig S. Gaide O. Martinon F. (2003). Two adjacent trimeric Fas ligands are required for Fas signaling and formation of a death-inducing signaling complex. *Mol. Cell Biol.* 23 1428–1440. 10.1128/mcb.23.4.1428-1440.2003 12556501 PMC141146

[B43] Hoogwater F. J. Nijkamp M. W. Smakman N. Steller E. J. Emmink B. L. Westendorp B. F. (2010). Oncogenic K-Ras turns death receptors into metastasis-promoting receptors in human and mouse colorectal cancer cells. *Gastroenterology* 138 2357–2367. 10.1053/j.gastro.2010.02.046 20188103

[B44] Huang B. Eberstadt M. Olejniczak E. T. Meadows R. P. Fesik S. W. (1996). NMR structure and mutagenesis of the Fas (APO-1/CD95) death domain. *Nature* 384 638–641. 10.1038/384638a0 8967952

[B45] Hughes M. A. Powley I. R. Jukes-Jones R. Horn S. Feoktistova M. Fairall L. (2016). Co-operative and hierarchical binding of c-FLIP and caspase-8: a unified model defines how c-FLIP isoforms differentially control cell fate. *Mol. Cell* 61 834–849. 10.1016/j.molcel.2016.02.023 26990987 PMC4819448

[B46] Itoh N. Yonehara S. Ishii A. Yonehara M. Mizushima S.-I. Sameshima M. (1991). The polypeptide encoded by the cDNA for human cell surface antigen Fas can mediate apoptosis. *Cell* 66 233–243. 10.1016/0092-8674(91)90614-5 1713127

[B47] Katoh K. Standley D. M. (2013). MAFFT multiple sequence alignment software version 7: improvements in performance and usability. *Mol. Biol. Evol.* 30 772–780. 10.1093/molbev/mst010 23329690 PMC3603318

[B48] Keppler O. T. Peter M. E. Hinderlich S. Moldenhauer G. Stehling P. Schmitz I. (1999). Differential sialylation of cell surface glycoconjugates in a human B lymphoma cell line regulates susceptibility for CD95 (APO-1/Fas)-mediated apoptosis and for infection by a lymphotropic virus. *Glycobiology* 9 557–569. 10.1093/glycob/9.6.557 10336988

[B49] Kischkel F. C. Hellbardt S. Behrmann I. Germer M. Pawlita M. Krammer P. H. (1995). Cytotoxicity-dependent APO-1 (Fas/CD95)-associated proteins form a death-inducing signaling complex (DISC) with the receptor. *EMBO J.* 14 5579–5588. 10.1002/j.1460-2075.1995.tb00245.x 8521815 PMC394672

[B50] Kleber S. Sancho-Martinez I. Wiestler B. Beisel A. Gieffers C. Hill O. (2008). Yes and PI3K bind CD95 to signal invasion of glioblastoma. *Cancer Cell* 13 235–248. 10.1016/j.ccr.2008.02.003 18328427

[B51] Kreuz S. Siegmund D. Rumpf J. J. Samel D. Leverkus M. Janssen O. (2004). NFkappaB activation by Fas is mediated through FADD, caspase-8, and RIP and is inhibited by FLIP. *J. Cell Biol.* 166 369–380. 10.1083/jcb.200401036 15289496 PMC2172264

[B52] Krissinel E. Henrick K. (2007). Inference of macromolecular assemblies from crystalline state. *J. Mol. Biol.* 372 774–797. 10.1016/j.jmb.2007.05.022 17681537

[B53] Lee K. H. Feig C. Tchikov V. Schickel R. Hallas C. Schutze S. (2006). The role of receptor internalization in CD95 signaling. *EMBO J.* 25 1009–1023. 10.1038/sj.emboj.7601016 16498403 PMC1409734

[B54] Leon-Bollotte L. Subramaniam S. Cauvard O. Plenchette-Colas S. Paul C. Godard C. (2011). S-nitrosylation of the death receptor fas promotes fas ligand-mediated apoptosis in cancer cells. *Gastroenterology* 140 2009–2018. 10.1053/j.gastro.2011.02.053 21354149

[B55] Levoin N. (2017). Sketching of CD95 oligomers by in silico investigations. *Methods Mol. Biol.* 1557 153–171. 10.1007/978-1-4939-6780-3_15 28078591

[B56] Liu W. Ramagopal U. Cheng H. Bonanno J. B. Toro R. Bhosle R. (2016). Crystal structure of the complex of human FasL and its decoy receptor DcR3. *Structure* 24 2016–2023. 10.1016/j.str.2016.09.009 27806260

[B57] Malleter M. Tauzin S. Bessede A. Castellano R. Goubard A. Godey F. (2013). CD95L cell surface cleavage triggers a prometastatic signaling pathway in triple-negative breast cancer. *Cancer Res.* 73 6711–6721. 10.1158/0008-5472.CAN-13-1794 24072745

[B58] Marchesi A. Gao X. Adaixo R. Rheinberger J. Stahlberg H. Nimigean C. (2018). An iris diaphragm mechanism to gate a cyclic nucleotide-gated ion channel. *Nat. Commun.* 9:3978. 10.1038/s41467-018-06414-8 30266906 PMC6162275

[B59] McCarthy A. E. Yoshioka C. Mansoor S. E. (2019). Full-length P2X7 structures reveal how palmitoylation prevents channel desensitization. *Cell* 179 659–670.e13. 10.1016/j.cell.2019.09.017 31587896 PMC7053488

[B60] Micheau O. (2018). Regulation of TNF-related apoptosis-inducing ligand signaling by glycosylation. *Int. J. Mol. Sci.* 19:E715. 10.3390/ijms19030715 29498673 PMC5877576

[B61] Micheau O. Tschopp J. (2003). Induction of TNF receptor I-mediated apoptosis via two sequential signaling complexes. *Cell* 114 181–190. 10.1016/s0092-8674(03)00521-x 12887920

[B62] Monet M. Poet M. Tauzin S. Fouque A. Cophignon A. Lagadic-Gossmann D. (2016). The cleaved FAS ligand activates the Na(+)/H(+) exchanger NHE1 through Akt/ROCK1 to stimulate cell motility. *Sci. Rep.* 6:28008. 10.1038/srep28008 27302366 PMC4908414

[B63] Muppidi J. R. Siegel R. M. (2004). Ligand-independent redistribution of Fas (CD95) into lipid rafts mediates clonotypic T cell death. *Nat. Immunol.* 5 182–189. 10.1038/ni1024 14745445

[B64] Murzin A. G. (1998). How far divergent evolution goes in proteins. *Curr. Opin. Struct. Biol.* 8 380–387. 10.1016/s0959-440x(98)80073-0 9666335

[B65] O’ Reilly L. A. Tai L. Lee L. Kruse E. A. Grabow S. Fairlie W. D. (2009). Membrane-bound Fas ligand only is essential for Fas-induced apoptosis. *Nature* 461 659–663. 10.1038/nature08402 19794494 PMC2785124

[B66] Orlinick J. R. Elkon K. B. Chao M. V. (1997). Separate domains of the human fas ligand dictate self-association and receptor binding. *J. Biol. Chem.* 272 32221–32229. 10.1074/jbc.272.51.32221 9405425

[B67] Pan L. Fu T. M. Zhao W. Zhao L. Chen W. Qiu C. (2019). Higher-order clustering of the transmembrane anchor of DR5 drives signaling. *Cell* 176 1477–1489.e14. 10.1016/j.cell.2019.02.001 30827683 PMC6529188

[B68] Papoff G. Cascino I. Eramo A. Starace G. Lynch D. H. Ruberti G. (1996). An N-terminal domain shared by Fas/Apo-1 (CD95) soluble variants prevents cell death in vitro. *J. Immunol.* 156 4622–4630. 8648105

[B69] Papoff G. Hausler P. Eramo A. Pagano M. G. Di Leve G. Signore A. (1999). Identification and characterization of a ligand-independent oligomerization domain in the extracellular region of the CD95 death receptor. *J. Biol. Chem.* 274 38241–38250. 10.1074/jbc.274.53.38241 10608899

[B70] Perez O. D. Kinoshita S. Hitoshi Y. Payan D. G. Kitamura T. Nolan G. P. (2002). Activation of the PKB/AKT pathway by ICAM-2. *Immunity* 16 51–65. 10.1016/s1074-7613(02)00266-2 11825565

[B71] Peter M. E. Hellbardt S. Schwartz-Albiez R. Westendorp M. O. Walczak H. Moldenhauer G. (1995). Cell surface sialylation plays a role in modulating sensitivity towards APO-1-mediated apoptotic cell death. *Cell Death Differ.* 2 163–171. 17180039

[B72] Poissonnier A. Sanseau D. Le Gallo M. Malleter M. Levoin N. Viel R. (2016). CD95-mediated calcium signaling promotes T helper 17 trafficking to inflamed organs in lupus-prone mice. *Immunity* 45 209–223. 10.1016/j.immuni.2016.06.028 27438772 PMC4961226

[B73] Putney L. K. Denker S. P. Barber D. L. (2002). The changing face of the Na+/H+ exchanger, NHE1: structure, regulation, and cellular actions. *Annu. Rev. Pharmacol. Toxicol.* 42 527–552. 10.1146/annurev.pharmtox.42.092001.143801 11807182

[B74] Qiao L. Studer E. Leach K. Mckinstry R. Gupta S. Decker R. (2001). Deoxycholic acid (DCA) causes ligand-independent activation of epidermal growth factor receptor (EGFR) and FAS receptor in primary hepatocytes: inhibition of EGFR/mitogen-activated protein kinase-signaling module enhances DCA-induced apoptosis. *Mol. Biol. Cell.* 12 2629–2645. 10.1091/mbc.12.9.2629 11553704 PMC59700

[B75] Reinehr R. Graf D. Haussinger D. (2003a). Bile salt-induced hepatocyte apoptosis involves epidermal growth factor receptor-dependent CD95 tyrosine phosphorylation. *Gastroenterology* 125 839–853. 10.1016/s0016-5085(03)01055-2 12949729

[B76] Reinehr R. Schliess F. Haussinger D. (2003b). Hyperosmolarity and CD95L trigger CD95/EGF receptor association and tyrosine phosphorylation of CD95 as prerequisites for CD95 membrane trafficking and DISC formation. *FASEB J.* 17 731–733. 10.1096/fj.02-0915fje 12586732

[B77] Reinehr R. Sommerfeld A. Haussinger D. (2008). CD95 ligand is a proliferative and antiapoptotic signal in quiescent hepatic stellate cells. *Gastroenterology* 134 1494–1506. 10.1053/j.gastro.2008.02.021 18471522

[B78] Sato T. Irie S. Kitada S. Reed J. C. (1995). FAP-1: a protein tyrosine phosphatase that associates with Fas. *Science* 268 411–415. 10.1126/science.7536343 7536343

[B79] Schneider P. Bodmer J. L. Holler N. Mattmann C. Scuderi P. Terskikh A. (1997). Characterization of Fas (Apo-1, CD95)-Fas ligand interaction. *J. Biol. Chem.* 272 18827–18833. 10.1074/jbc.272.30.18827 9228058

[B80] Schneider P. Holler N. Bodmer J. L. Hahne M. Frei K. Fontana A. (1998). Conversion of membrane-bound Fas(CD95) ligand to its soluble form is associated with downregulation of its proapoptotic activity and loss of liver toxicity. *J. Exp. Med.* 187 1205–1213. 10.1084/jem.187.8.1205 9547332 PMC2212219

[B81] Schneidman-Duhovny D. Inbar Y. Nussinov R. Wolfson H. J. (2005a). Geometry-based flexible and symmetric protein docking. *Proteins* 60 224–231. 10.1002/prot.20562 15981269

[B82] Schneidman-Duhovny D. Inbar Y. Nussinov R. Wolfson H. J. (2005b). PatchDock and SymmDock: servers for rigid and symmetric docking. *Nucleic Acids Res.* 33 W363–W367. 15980490 10.1093/nar/gki481 PMC1160241

[B83] Scott F. L. Stec B. Pop C. Dobaczewska M. K. Lee J. J. Monosov E. (2009). The Fas-FADD death domain complex structure unravels signalling by receptor clustering. *Nature* 457 1019–1022. 10.1038/nature07606 19118384 PMC2661029

[B84] Shatnyeva O. M. Kubarenko A. V. Weber C. E. Pappa A. Schwartz-Albiez R. Weber A. N. (2011). Modulation of the CD95-induced apoptosis: the role of CD95 N-glycosylation. *PLoS One* 6:e19927. 10.1371/journal.pone.0019927 21625644 PMC3097226

[B85] Siegel R. M. Frederiksen J. K. Zacharias D. A. Chan F. K. Johnson M. Lynch D. (2000). Fas preassociation required for apoptosis signaling and dominant inhibition by pathogenic mutations. *Science* 288 2354–2357. 10.1126/science.288.5475.2354 10875918

[B86] Siegmund D. Lang I. Wajant H. (2017). Cell death-independent activities of the death receptors CD95, TRAILR1, and TRAILR2. *FEBS J.* 284 1131–1159. 10.1111/febs.13968 27865080

[B87] Steinbach J. P. Supra P. Huang H. J. Cavenee W. K. Weller M. (2002). CD95-mediated apoptosis of human glioma cells: modulation by epidermal growth factor receptor activity. *Brain Pathol.* 12 12–20. 10.1111/j.1750-3639.2002.tb00418.x 11770895 PMC8095827

[B88] Suda T. Hashimoto H. Tanaka M. Ochi T. Nagata S. (1997). Membrane Fas ligand kills human peripheral blood T lymphocytes, and soluble Fas ligand blocks the killing. *J. Exp. Med.* 186 2045–2050. 10.1084/jem.186.12.2045 9396774 PMC2199173

[B89] Tanaka M. Itai T. Adachi M. Nagata S. (1998). Downregulation of Fas ligand by shedding. *Nat. Med.* 4 31–36. 10.1038/nm0198-031 9427603

[B90] Tanaka M. Suda T. Haze K. Nakamura N. Sato K. Kimura F. (1996). Fas ligand in human serum. *Nat. Med.* 2 317–322. 8612231 10.1038/nm0396-317

[B91] Tauzin S. Chaigne-Delalande B. Selva E. Khadra N. Daburon S. Contin-Bordes C. (2011). The naturally processed CD95L elicits a c-yes/calcium/PI3K-driven cell migration pathway. *PLoS Biol.* 9:e1001090. 10.1371/journal.pbio.1001090 21713032 PMC3119658

[B92] Tauzin S. Debure L. Moreau J. F. Legembre P. (2012). CD95-mediated cell signaling in cancer: mutations and post-translational modulations. *Cell. Mol. Life Sci.* 69 1261–1277. 10.1007/s00018-011-0866-4 22042271 PMC11115069

[B93] Voss M. Lettau M. Paulsen M. Janssen O. (2008). Posttranslational regulation of Fas ligand function. *Cell Commun. Signal.* 6:11. 10.1186/1478-811X-6-11 19114018 PMC2647539

[B94] Wagner K. W. Punnoose E. A. Januario T. Lawrence D. A. Pitti R. M. Lancaster K. (2007). Death-receptor O-glycosylation controls tumor-cell sensitivity to the proapoptotic ligand Apo2L/TRAIL. *Nat. Med.* 13 1070–1077. 10.1038/nm1627 17767167

[B95] Wajant H. (2015). Principles of antibody-mediated TNF receptor activation. *Cell Death Differ.* 22 1727–1741. 10.1038/cdd.2015.109 26292758 PMC4648319

[B96] Wako H. Endo S. (2017). Normal mode analysis as a method to derive protein dynamics information from the protein data bank. *Biophys. Rev.* 9 877–893. 10.1007/s12551-017-0330-2 29103094 PMC5711701

[B97] Wang L. Yang J. K. Kabaleeswaran V. Rice A. J. Cruz A. C. Park A. Y. (2010). The Fas-FADD death domain complex structure reveals the basis of DISC assembly and disease mutations. *Nat. Struct. Mol. Biol.* 17 1324–1329. 10.1038/nsmb.1920 20935634 PMC2988912

[B98] Wang X. Defrances M. C. Dai Y. Pediaditakis P. Johnson C. Bell A. (2002). A mechanism of cell survival: sequestration of Fas by the HGF receptor Met. *Mol. Cell* 9 411–421. 11864613 10.1016/s1097-2765(02)00439-2

[B99] Wang Y. Bugge K. Kragelund B. B. Lindorff-Larsen K. (2018). Role of protein dynamics in transmembrane receptor signalling. *Curr. Opin. Struct. Biol.* 48 74–82. 10.1016/j.sbi.2017.10.017 29136528

[B100] Wick W. Fricke H. Junge K. Kobyakov G. Martens T. Heese O. (2014). A phase II, randomized, study of weekly APG101+reirradiation versus reirradiation in progressive glioblastoma. *Clin. Cancer Res.* 20 6304–6313. 10.1158/1078-0432.CCR-14-0951-T 25338498

[B101] Yoder N. Yoshioka C. Gouaux E. (2018). Gating mechanisms of acid-sensing ion channels. *Nature* 555 397–401. 10.1038/nature25782 29513651 PMC5966032

